# Risk Factors for Foot Ulcers in Patients with Diabetes Mellitus - A Short Report from Vellore, South India

**DOI:** 10.4103/0970-0218.62582

**Published:** 2010-01

**Authors:** Reginald Alex, Benjamin Ratnaraj, Blessed Winston, D Nathaniel Samson Devakiruba, Clarence Samuel, Jacob John, Venkata Raghava Mohan, Jasmine Helan Prasad, KS Jacob

**Affiliations:** Department of Community Health, Christian Medical College, Vellore - 632 002, India; 1 Department of Psychiatry, Christian Medical College, Vellore - 632 002, India

## Introduction

Diabetes mellitus, a metabolic disease, has a population prevalence of about 10-15%. The incidence of foot ulcers range from 8 to 17% in the cohort studies, with varying lengths of follow-up, and cause severe disability and possible hospitalization to patients and considerable economic burden to families.(1–4) A variety of foot lesions are seen in people with uncontrolled diabetes mellitus namely fissures, abscess, cellulites, ulcers, claw toes and Charcot's joints. There is a risk of developing gangrene and of consequent amputation of the foot especially for people from the lower socioeconomic strata and for those living in rural areas. Clinical guidelines recommend that all patients with diabetes should be screened annually to establish their risk of foot ulceration.(2) Diagnostic tests and physical signs that detect peripheral neuropathy (biothesiometry, monofilaments and absent ankle reflexes), and those that detect excessive plantar pressure (peak plantar pressure and joint deformity) were all significantly associated with future diabetic foot ulceration. However, there was a paucity of evidence from India concerning the predictive value of symptoms and signs.

This study aimed to examine the risk factors for foot ulcers in patients with diabetes mellitus attending the Community Health and Development (CHAD) Hospital, Christian Medical College, Vellore, a secondary care facility.

## Materials and Methods

### Setting

The Community Health and Development Hospital, Vellore, is a 120-bed hospital. It is the base hospital of a comprehensive community health program for Kaniyambadi block with an estimated population of 110 000 and a geographical area of 182 sq. km. The hospital treats a wide variety of patients including those with tuberculosis, leprosy, rheumatic heart disease and AIDS. Antenatal, sterilization, child health clinics and deliveries form a major component of health care. All patients with diagnosed to have diabetes mellitus in CHAD's general health and peripheral clinics are referred to the diabetic clinic. The diabetic clinic is conducted weekly and the services of an internal medicine specialist, an ophthalmologist, a nurse trained in diabetic care, a physiotherapist, a foot care therapist and a cobbler are available. Patients who attend the diabetic special clinic are given dietary advice, education on foot care and exercise. Blood tests and examination of eyes are done periodically.

### Database

Data on socio-demographic characteristics, clinical details related to diabetes, hypertension and obesity, details of foot ulcers, deformity, neuropathy and peripheral pulses are collected routinely. Data on all cases attending this clinic is computerized.

### Sample

All patients registered from September 2005 to May 2006 were included in the study. All patients with diabetes mellitus who were on treatment and had at least one episode of foot ulcer during the course of their treatment were considered as ‘cases’. Patients with other causes of neuropathy like leprosy were excluded. The first 45 patients from diabetic register who did not have foot ulcers were selected as ‘controls’.

Ulcer was defined as full thickness skin break below the level of malleoli. Neuropathy was defined as inability of patients to detect 10 g monofilaments on more than one site of 10 g on the plantar aspect of both feet.(1) The 10-g monofilament was tested on five plantar sites on each foot. These were the 1^st^, 2^nd^, 3^rd^ and 5^th^ metatarsal head and the great toe. The monofilament is applied to the site applying a pressure where the filament was just able to bend. ‘Absent pulses’ was defined as absence of both dorsalis pedis and posterior tibial pulse in either foot.(1) Foot deformity was defined as change in foot shape assessed subjectively due to the primary disease or amputation.(1) Previous ulceration was identified by reading case notes and asking the patient.(1) Physical disability was defined as the patients not being able to reach their feet, whilst visual disability is defined as patients not being able to see their feet safely enough to cut their nails as judged by the clinician.(1)

### Statistics

Descriptive statistics were calculated for continuous variables while frequency distributions were obtained for categorical variables. The Chi-square test was employed to assess the significance of the association between categorical variables and the presence/absence of foot ulcers. Univariate odds ratios were calculated, and adjusted odds ratios obtained using logistic regression after adjusting for age and gender.

## Results

Forty five patients with foot ulcers were treated at the hospital during the one-year study period. Forty five controls attending the same diabetic clinic during the same period were also selected for the analysis. The mean age of the total sample was 55.5 (SD 11.6) years. The majority of the sample was male (51.1%), non-smokers (95.6%) and did not have hypertension (67.8%). The average duration after diagnosis of diabetes mellitus was 6.1 (SD 6.3) years. The majority did not have peripheral neuropathy (81.1%), absent peripheral pulses (90.0%), pre-ulcerous states (90.0%), callous (89.9%), fissures on feet (64.4%), nail pathology (97.1%), foot deformity (93.3%) or disability (94.4%). The majority were on treatment with diet and oral anti-diabetic medication (90.0%).

The odds ratios and adjusted odds ratio are shown in Table 1. Patients with diabetes on insulin replacement were at a greater risk for the development of foot ulcers as compared to those on diet and oral anti-diabetic medication. Similarly, people with peripheral neuropathy were also at increased risk. However, those with hypertension seem to be protected. All patients with hypertension in the sample were on ACE inhibitors (Enalapril). The increased risk remained after adjustment for age and gender using logistic regression. The presence of callous, pre-ulcers and deformity were also related to the presence of foot ulcers among those with diabetes.

**Table 1:** Risk factors for developing foot ulcers in patients with diabetes mellitus

Characteristic	Cases *n*(%)	Controls *n*(%)	Univariate statistics		Multivariate statistics	
				
			Odds ratio (95% CI)	*P* value	Adjusted odds ratios (95% CI)^1^	*P* value
Gender- Male	22 (48.9)	24 (53.3)	0.84 (0.37-1.91)	0.673	0.83 (0.36-1.90)	0.652
Age- Over 55 years(2)	25 (55.6)	23 (51.1)	1.20 (0.52-2.74)	0.673	1.21 (0.53-2.78)	0.652
Body mass index >25(2)	24 (54.5)	26 (57.8)	0.88 (0.38-2.03)	0.759	1.27 (0.55-2.95)	0.578
Hypertension on treatment with ACEI	10 (22.2)	19 (42.2)	0.39 (0.16-0.98)	0.042	0.29 (0.10-0.80)	0.018
Smoker	2 (4.4)	1 (2.3)	2.00 (0.18-22.89)	0.570	2.47 (0.21-29.76)	0.477
Duration of diabetes in years >3(2)	28 (62.2)	22 (48.9)	1.72 (0.74-3.99)	0.203	1.20 (0.52-2.78)	0.669
Treated with anti-hyperglycemic medication or insulin	33 (82.5)	26 (66.7)	2.36 (0.82-6.76)	0.106	2.39 (0.82-6.92)	0.11
Treated with insulin	8 (17.8)	1 (2.2)	9.51 (1.14-79.60)	0.014	11.05 (1.29-94.54)	0.028
Nail pathology present	1 (2.2)	1 (2.2)	1.00 (0.61-16.50)	1.000	1.00 (0.06-16.57)	0.998
Fissures present	13 (28.9)	19 (42.2)	0.56 (0.23-1.33)	0.186	0.52 (0.21-1.29)	0.157
Callus present	2 (4.4)	8 (17.8)	0.22 (0.43-1.08)	0.044	0.21 (0.42-1.08)	0.061
Disability present	3 (6.7)	2 (4.4)	1.27 (0.24-9.66)	0.645	1.49 (0.24-9.39)	0.674
Pre-ulcer present	9 (20.0)	0 (0.0)	-	0.002	-	-
Deformity present	6 (13.3)	0 (0.0)	-	0.011	-	-
Absent pulses	6 (13.3)	2 (4.4)	3.23 (0.62-16.97)	0.147	2.99 (0.55-16.21)	0.205
Peripheral neuropathy present	15 (33.3)	2 (4.4)	10.75 (2.29-50.51)	0.000	10.86(2.28-51.77)	0.003

1 Adjusted for age and gender using logistic regression. Median values used to divide the sample

## Discussion

Foot ulcers is a disabling complication and not uncommon among people with diabetes mellitus. The disability and possible progression to the loss (amputation) of digits and limbs make it a serious issue.(1,2) This study attempted to examine the risk factors for foot ulceration using a case control design. Systematic assessments done routinely in the special clinic and the computerization of the data were the strengths of the study. Assessment of arterial pulses using Doppler and biothesiometer were not practical and cost effective in secondary care clinical practice and hence were not used in this study.(1) However, assessment using Doppler often give a misleading ankle/brachial index (ABI) in patients with diabetes due to arterial calcification. Foot pulses were used in the clinical assessment, and their absence is usually associated with an ABI of <0.76.(1)

The risk factors identified included the need for insulin therapy for uncontrolled blood sugars possibly reflecting a severe form of the condition with poorer glycemic control. The presence of peripheral neuropathy seems to contribute to the development of ulceration and those with pre-ulceration, callosities and deformity seem to be at increased risk. However, those with hypertension seem to be protected. As all the patients in the study were on ACE inhibitors, a possible mechanism for such protection could be due to improved endothelial function leading to enhanced peripheral circulation.(5,6) People with the presence of these risk factors will require greater care in preventing such ulceration. Available international literature is supportive of these risk factors as causative for foot ulcers in people with diabetes.(1–4)

Routine foot care should be available to every patient with diabetes ideally, but the reality of most healthcare facilities in India lack resources resulting in the absence of such care. Efficient use of resources dictates that routine podiatry care should be directed towards patients in greatest need. Unfortunately, this is often not the case, with needy patients being denied timely access to podiatry and foot care, because specialist clinics are overbooked with patients with lesser risk. Diagnostic tests and clinical signs are helpful in predicting the risk of diabetic foot ulceration(1) and are more cost effective in many smaller healthcare facilities. This study adds to the limited data from India. Further studies are needed to assess the independent predictive value of all elements of patient history, physical signs and diagnostic tests when assessing the risk of diabetic foot ulceration.
